# The Cause of Drunkenness among Women

**Published:** 1898-07-16

**Authors:** 


### THE CAUSE OF DRUNKENNESS AMONG WOMEN.

In giving evidence before the Liquor Commission, Mr. Lawson Tait drew a marked distinction between drunkards of the two sexes, and said that a drunken woman required special treatment. Almost without exception, he had been able to trace the cases of female drunkenness to physical or mental suffering. Female drunkards occurred, he said, in all classes of society, even in the highest, and in analysing a set of 108 cases, he found they fell into two groups—(1) a small one of 12 persons, having an average age of 26, and (2) a large one of 96, with an average age of 48—and he drew the conclusion that the suffering due to the change of life was the cause of the second group, and that when the period was passed the tendency ceased. He thought also that in the first group the drunkenness was due to physical suffering, and that when the cause was removed the drunkenness diminished. To treat such cases, then, in inebriate homes was fundamentally wrong in the case of women; nay, he went further, saying that complete deprivation of drink should not necessarily be a part of their treatment, and that alcohol should rather be used as an article of diet to help them over their trouble. That the pains and "sinkings" felt by many women in response to disturbances of uterine and ovarian function very commonly lead them to habits of alcoholic excess no one can doubt, but neither can we doubt that, in the presence of these distressing sensations, the tendency to give way to the temptation is much increased by the facilities offered for obtaining drink, so that we can hardly agree that the abolition of these facilities, grocers' licences, &c., would be quite inoperative in stopping the evil, although we may admit that, when once a woman has given way to the habit, the drying-up of one source of supply is not at all likely to check her downward progress. Without doubt the proper thing is to remove the cause of the craving. We must not, however, be too sure that a habit will be stopped by the removal of the cause of its beginning. The removal of the temptation to its continuance is likely also to be of use.

